# Potential neuropsychological mechanism involved in the transition from suicide ideation to action – a resting-state fMRI study implicating the insula

**DOI:** 10.1192/j.eurpsy.2023.2444

**Published:** 2023-09-11

**Authors:** Shulin Fang, Samuel F. Law, Xinlei Ji, Qinyu Liu, Panwen Zhang, Runqing Zhong, Huanhuan Li, Xiaosheng Wang, Shuqiao Yao, Xiang Wang

**Affiliations:** 1 Medical Psychological Center, The Second Xiangya Hospital, Central South University, Changsha, Hunan, China; 2 China National Clinical Research Center on Mental Disorders (Xiangya), Changsha, Hunan, China; 3 Department of Psychiatry, University of Toronto, Toronto, Ontario, Canada; 4 Shanghai Songjiang Jiuting Middle School, Shanghai, China; 5 Department of Psychology, Renmin University of China, Beijing, China; 6 Department of Human Anatomy and Neurobiology, Xiangya School of Medicine, Central South University, Hunan, China

**Keywords:** decision-making, ideation-to-action framework, insula, resting-state fMRI, suicide

## Abstract

**Background:**

Understanding the neural mechanism underlying the transition from suicidal ideation to action is crucial but remains unclear. To explore this mechanism, we combined resting-state functional connectivity (rsFC) and computational modeling to investigate differences between those who attempted suicide(SA) and those who hold only high levels of suicidal ideation(HSI).

**Methods:**

A total of 120 MDD patients were categorized into SA group (n=47) and HSI group (n=73). All participants completed a resting-state functional MRI scan, with three subregions of the insula and the dorsal anterior cingulate cortex (dACC) being chosen as the region of interest (ROI) in seed-to-voxel analyses. Additionally, 86 participants completed the balloon analogue risk task (BART), and a five-parameter Bayesian modeling of BART was estimated.

**Results:**

In the SA group, the FC between the ventral anterior insula (vAI) and the superior/middle frontal gyrus (vAI-SFG, vAI-MFG), as well as the FC between posterior insula (pI) and MFG (pI-MFG), were lower than those in HSI group. The correlation analysis showed a negative correlation between the FC of vAI-SFG and psychological pain avoidance in SA group, whereas a positive correlation in HSI group. Furthermore, the FC of vAI-MFG displayed a negative correlation with loss aversion in SA group, while a positive correlation was found with psychological pain avoidance in HSI group.

**Conclusion:**

In current study, two distinct neural mechanisms were identified in the insula which involving in the progression from suicidal ideation to action. Dysfunction in vAI FCs may gradually stabilize as individuals experience heightened psychological pain, and a shift from positive to negative correlation patterns of vAI-MFC may indicate a transition from state to trait impairment. Additionally, the dysfunction in PI FC may lead to a lowered threshold for suicide by blunting the perception of physical harm.

## Introduction

Suicide is a complex biopsychosocial phenomenon, resulting in over 700,000 deaths a year [1]. Mental illness is involved in more than 90% of people who die by suicide [2]. Major depressive disorder (MDD) is the most prevalent contributing psychiatric diagnosis, with a lifetime prevalence of suicide attempts at 31% [3], and is related to 30% of all suicide deaths [4].

The mechanisms of how MDD influences suicide remain unclear, and our ability to predict suicide behavior remains poor [5]. Difficulty in distinguishing individuals with MDD who only have suicide ideation from those who carry out actual suicide behavior is a key challenge. Notably, previous large cohort studies and meta-analyses have provided evidence that MDD may only affect suicide ideation and not suicide behavior [5–7]. There is increasing evidence that suggests suicide behavior is potentially an independent behavior syndrome [8], as outlined in the current Diagnostic and Statistical Manual (DSM-5) [9].

Research also demonstrates that suicide ideation and suicide behavior involve distinct risk factors and psychological mechanisms. For example, risk factors including depression, hopelessness, and even impulsivity are more associated with suicide ideation than suicide behavior [10, 11]. Based on the above, Klonsky and colleagues have proposed a suicide “ideation-to-action” framework to focus on the mechanisms involved in the transition from suicide ideation to suicide behavior [12].

The transition from suicide ideation to action is known to be associated with impaired decision-making, with likely unique characteristics and biases [13–15]. More specifically, when compared to people without a history of suicide attempts or healthy controls, suicide attempters have more negative evaluations of the future, and paradoxically more aversion to loss and risk [16], suggesting that they may choose suicide as a way to solve current dilemmas and stress to get immediate “reward” outcome [17]. The study of the ideation-to-action transition is complex. One novel method, the balloon analogue risk task (BART), with established high ecological validity that simulates real-world decision-making situations through sequential risk-taking choices [18], and the ability to study the underlying cognitive processes using multi-parametric computational models, could be uniquely suited to study such suicide-related decision-making [19–21].

Psychological pain is also known to play an important role in the transition from suicide ideation to action [22, 23]. Li and colleagues divided psychological pain into three different components, namely psychological pain arousal, psychological painful feelings, and psychological pain avoidance [24]. Various studies, including a recent report using machine learning methods, have found that only the psychological pain avoidance component predicted suicide action and distinguished suicide ideation from suicide action [24–26].

Resting-state functional MRI (rs-fMRI) can be a useful tool to probe the neural mechanisms involved in the transition from suicide ideation to action as it investigates the actual state in which such a decision is made. Research has shown that people with a high level of suicide ideation usually repeatedly consider the significance of their life, and the consequences of suicide, and run through the decision-making process of whether to carry out suicide in a “resting state” [27]. While it is difficult to directly study those who completed suicide, it may be possible to identify potential neural mechanisms involved in the transition from suicide ideation to suicide behavior by comparing the rs-fMRI functional connectivities in the resting states between suicide ideators and suicide attempters. Employing this approach, Wagner and colleagues’ pioneering study has found abnormalities in the frontal–parietal network and some subcortical areas distinguishing between suicide ideators and suicide attempters [28]. However, this study did not match the levels of suicide ideation between the two groups, making it impossible to conclude if the findings stem from differences in levels of suicide ideation, suicide behavior, or both.

The current rs-fMRI study aims to further this line of research with a number of innovations. Firstly, we focus the current study on the insula and the dorsal anterior cingulate cortex (dACC) as our regions of interest (ROI), and locales for seed-based correlation analysis on functional connectivity for the whole brain. These chosen ROIs are informed by Schmaal and colleagues’ proposal regarding “a tentative brain model of suicidal thoughts and behaviors (STBs)” that involves multiple brain circuitries, particularly the prefrontal cortex, insula, and the dACC, among others. Their model highlights that the generation of suicidal ideation and action are related to excessive negative internal states, negative self-referencing, impairments in future thinking, emotional regulations, and decision-making [29]. The chosen ROIs are also thought to likely play a role in mediating the transition from suicidal ideation to action through their involvement in the bottom-up and top-down systems. It is also known that these ROIs are key components of the salience network, playing important roles in mediating or switching between the default mode network/emotional mode network and the cognitive mode network [30, 31]. Secondly, we have a relatively large, matched sample size. Thirdly, we rigorously controlled the level of suicide ideation between the two groups. Finally, we have employed correlation analysis to explore the relationship between functional connectivity and established clinical and psychological variables that could distinguish suicide ideators from suicide attempters, including parameters from the computational model of BART.

## Method

### Participant recruitment, psychological measurements, and group assignment

From the outpatient departments of the Second Xiangya Hospital, a general hospital in Changsha, Hunan, China, we recruited 120 patients with major depressive disorder (MDD).

The inclusion criteria were (a) diagnosis of MDD using the structured clinical interview for DSM-TR Axis I Disorders- Patient Edition (SCID-P) [32]; (b) BDI score greater than or equal to 19; (c) BSI-C higher or equal to 2, and BSI-W higher or equal to 16 (both help to define a high level of suicidal ideation according to guidelines of Beck and Steer) [33]; and (d) age between 16 and 45 years. The exclusion criteria were (a) diagnosis of other Axis I disorders (e.g., attention deficit hyperactivity disorder, substance use disorders); (b) history of severe head trauma or major physical illness; (c) having metallic objects in the body*;* and (d) history of major interventions affecting brain functions (e.g., electroconvulsive therapy, transcranial magnetic stimulation therapy, and ketamine treatment). Patients who had no suicide attempt were assigned to the high suicidal ideation group (HSI, N = 73), and those with high ideation and suicide attempt history to the suicide attempt group (SA, N = 47).

Participants’ history of suicide attempts was assessed by two psychiatrists, guided by the Colombian Suicide Severity Rating Scale (C-SSRS) [34], based on interviews, medical records, and information provided by family and friends. The term “suicide attempt” was operationally defined as a deliberate act of self-harm with the purpose of ending one’s own life, wherein a minimum duration of 15 minutes elapsed between the decision to commit suicide and its actual execution, thereby excluding impulsive acts of suicide [35]. Within the group of individuals who attempted suicide (SA), we conducted a thorough assessment of the methods employed for the most recent attempt. Specifically, we found wrist cutting in 19 patients, overdose of medication in 12 patients, jumping from a building or river in 11 patients, traffic collision in 3 patients, burning charcoal in 1 patient, and hanging in 1 patient. According to the global impression of lethality item of the Scale for Assessment of Lethality of Suicide Attempt (SALSA) [36], all types of suicide attempts were found to be moderately to severely lethal. The detailed methods and frequency are provided in the Supplementary Materials(Supplementary Table S1). The mean length of time since the most recent suicide attempt was 13.16 months, and 37 out of 47 participants met the criteria for suicidal behavioral disorders in DSM-5 [9] whose most recent suicide attempt occurred within the last two years.

This study was approved by the Ethics Committee of the Second Xiangya Hospital of Central South University. All participants were thoroughly informed of the content and risks of the experiment and signed a consent form.

## Measures

### Beck depression inventory (BDI)

The BDI-I developed by Beck was used to assess the level of depression in the past week [37]. The inventory consists of 21 items on a 4-point scale (0–3). The higher the score, the higher the level of depression. The revised Chinese version was used in this study and showed good reliability and validity (Cronbach’s α = 0.89) in a depressed sample [38].

### State–trait inventory (STAI)

The STAI is used to assess anxiety on both state and trait dimensions [39]. The scale consists of 40 items on a 4-point scale (1–4) with 20 items for each dimension. A revised Chinese version of the STAI was used in this study [40], which showed good reliability and validity in a sample of college students (Cronbach’s α = 0.91/0.88).

### Beck suicidal ideation scale (BSI)

The Beck Suicide Ideation Scale (BSI), developed by Beck and Steer, is utilized to assess the levels of suicidal ideation in individuals during the current week (BSI-C) and their worst periods (BSI-W) [33]. This scale consists of 19 items, each rated on a 3-point scale ranging from 0 to 2 (None, slightly, moderately to strongly). Higher scores indicate higher levels of suicidal ideation. Only those who answered slightly or moderately to strongly on item 4 or item 5 were eligible to complete the entire scale (i.e., wish to commit suicide, and desire for the passive suicide attempt, respectively). For the purposes of this study, the Chinese version of the BSI was translated and revised by the Beijing Suicide Research and Prevention Center. The Chinese version showed good reliability, with an internal consistency coefficient of 0.96 for the BSI-C and 0.94 for the BSI-W [41].

### Three-dimensional psychological pain scale (TDPPS)

The TDPPS is a 21-item scale used to assess psychological pain in three dimensions: cognitive, affective, and motivational [24]. The cognitive dimension, namely psychological pain arousal, measures psychological pain generated by memories of traumatic experiences. The affective dimension, namely psychological pain feelings, measures feelings of pain and physical reactions. The motivational dimension, namely psychological pain avoidance, measures the intensity of suicide as a means of escaping intolerable psychological pain. The scale is based on a 5-point scale (1–5), with higher scores indicating higher levels of psychological pain. This scale showed good reliability and validity both in the sample of college students (Cronbach’s α = 0.89 ~ 0.91) and in depression disorder (Cronbach’s α = 0.68 ~ 0.69).

### The balloon analogue risk task (BART)

The BART is a computer-based behavior task that measures risk-taking through sequential risk-taking choices [18]. Briefly summarizing, the participant is instructed to press button 1 to inflate a balloon or button 2 to discontinue inflation. The participants are rewarded if the series of balloons are inflated to be as big as possible with the least amount of bursting, repeated over 20 minutes [42]. A cumulative score keeps track of the reward for each inflated balloon, and deductions are made for exploded balloons. The balloons could explode at any size, and the reward and risk of explosion increased as the balloons increased in size. A total of 86 (out of 120) participants completed the BART. There were no significant differences in clinical measures between participants who completed BART and those who did not (Detailed in Supplementary Table S2).

### Behavior modeling using BART

Analyses of the behavioral indicators allow for further understanding of the participants’ decision-making process. We adopted the five-parameter model of BART based on the Exponential Weight Updating (EW) model and built a hierarchical Bayesian model using the hBayesDM tools [43] in the R platform (version 3.6.2). The EW model has exhibited good performance in suicide-related studies based on previous research [44]. The final five parameters were: ψ (prior belief of burst, index of the initial belief about the probability of exploding), ξ (updating exponent, index of how quickly participants update their beliefs based on observations), ρ (risk preference, index of tendency to avoid risk), τ (inverse temperature, index of the deterministic or random of the choice), and λ (loss aversion, index of the tendency to avoid loss) [21] (see Supplementary Materials for all BART and behavioral model-related details).

### Imaging acquisition and preprocessing

Imaging data were acquired using a Simens 3 T scanner (Skyra, Simens, Erlangen, Germany) with a 32-channel, high-resolution, transmit/receive brain volume coil. The functional data were acquired using a single-shot gradient echo-echo planar (EPI) imaging sequence, and high-resolution coplanar anatomical images were acquired using magnetization-prepared rapid gradient echo (MPRAGE). The imaging data were preprocessed using the conn toolbox (CONN 20b, HTTPS:// conn-toolbox.org/) implemented in MATLAB R2018a and applying standard preprocessing steps, including slice timing correction, realignment, normalization, smooth, and bandpass filter (see Supplementary Materials for details).

### Region of interest definition

The insula and the dorsal anterior cingulate cortex (dACC) were chosen as the regions of interest (ROI). Descriptors of the insula used in this study were based on the template proposed by Faillenot et al. [45], which divided the insula into three systems, including the ventral anterior insula (vAI), the dorsal anterior insula (dAI), and the posterior insula (pI) [46]. The definition of the dACC is based on the template proposed by Margulies et al. [47].

## Statistics analysis

### Clinical variables and BART parameters analysis

Demographic and clinical characteristics were compared using chi-square tests in the SPSS 25.0 package with a significance criterion of *p* < 0.05.

For BART parameter analyses, the Highest Density Interval (HDI) of the posterior distribution was used to compare the HSI and SA groups. HDI, also known as Bayesian Confidence Interval, describes the region of the posterior distribution of a parameter. When comparing the two samples, the HDI represents the difference interval of posterior distribution between the two groups. When 95% HDI excluded zero, we can reject the null hypothesis and consider the results significantly different between the two groups.

### Functional connectivity analysis

In our seed-based correlation analysis, the mean resting-state Blood Oxygen Level Dependent (BOLD) time series of each ROI and each voxel of other parts of the whole brain for each participant were extracted and the functional connectivity (FC) map between each ROI and the whole brain for each individual were then calculated. The obtained correlation coefficients were then transformed to normally distributed *z* scores using Fisher’s r-to-z transformation. A two-sample t-test of FC was conducted between the HSI group and the SA group with suicide ideation levels (including the current week point and the worst point) as covariates. The mean framewise displacement (FD) [48] and scan type were included as additional covariates in the two-sample t-test for FC. The two-sample t-test thresholds were set at *p* < 0.001 for uncorrected voxel levels and *p* < 0.05 for False Discovery Rate (FDR)-corrected cluster levels.

### Correlation analysis

Pearson’s correlation analyses between FC and all clinical and psychological variables were performed. The significance threshold was set at *p* < 0.05.

## Results

### Demographic, clinical, and psychological characteristics

As seen in Table 1, for demographic characteristics, except for the proportion of first episode, there was no significant difference between the HSI and SA groups in terms of age, sex, education, duration of illness, antidepressants, and history of suicidal self-injuries (all *p* > 0.05). The difference in depressive symptoms between the first episode of MDD (Mean = 35.78, SD = 7.03) and recurrent MDD (Mean = 38.47, SD = 7.32) was not significant (*t* = 1.41, *p* = 0.17).

**Table 1.** Comparing demographic, clinical, and psychological characteristics between HSI and SA groups

	HSI (73)	SA (n = 47)	*X*^2^/t	p	Cohen’d
Male/Female (n)	16/57	9/38	0.13	0.72	–
Age (years)	21.56 ± 5.07	20.96 ± 5.46	0.62	0.54	–
Education (years)	13.30 ± 2.87	13.53 ± 2.43	−0.46	0.65	–
Duration of illness (months)	13.84 ± 14.41	18.16 ± 17.81	−1.41	0.16	–
First episode/non-first episode	67/6	36/11	0.42	0.02	–
Antidepressant (yes/no)	15/58	13/34	0.81	0.37	–
Suicidal self-injurious behavior(with/without)	10/47	16/73	0.01	0.93	–
BDI score	35.41 ± 6.87	37.30 ± 7.37	−1.41	0.16	–
BSI_C score	11.41 ± 6.31	16.51 ± 7.38	−4.04	<0.01	0.76
BSI_W score	22.22 ± 4.54	28.64 ± 4.86	−7.36	<0.01	1.38
STAI score					
S_AI	60.22 ± 10.67	62.23 ± 10.38	−0.98	0.33	–
T_AI	65.26 ± 6.85	65.47 ± 6.68	−0.16	0.88	–
TDPPS score					
Total score	64.63 ± 8.37	67.15 ± 10.37	−1.46	0.15	–
Pain arousal	28.78 ± 4.83	29.21 ± 5.82	−0.44	0.66	–
Painful feelings	25.33 ± 3.54	25.57 ± 4.14	−0.35	0.73	–
Pain avoidance	10.52 ± 2.85	12.36 ± 2.48	−3.63	<0.01	0.68

Abbreviations: HSI, High suicide ideation group; SA, Suicide attempt group; BDI, Beck Depression Inventory; BSI_C, Beck Scale for Suicide Ideation at the current time; BSI_W, Beck Scale for Suicide Ideation at the worst time; STAI: State–trait Anxiety Inventory; SAI: State anxiety inventory; TAI: Trait Anxiety Inventory; TDPPS: Three-dimensional Psychological Pain Scale.

For clinical characteristics, the level of depression as measured by the BDI-I and anxiety as measured by the STAI were not significantly different between the HSI and SA groups (all *p* > 0.05). Both the level of suicide ideation at the current week (SA: range 2–28; HSI: range 2–31) and the worst time showed that the SA group (SA: range 16–35; HSI: range 17–36) was higher than the HSI group (all *p* < 0.01).

For psychological factors, the SA group was significantly higher than the HSI group (*p* < 0.01) in psychological pain avoidance, but no differences were found in psychological pain arousal, or painful feelings (*p* > 0.05).

### Parameters of BART

The HDI analysis showed that the SA group had higher λ (loss aversion) than the HSI group (95% HDI [0.3608, 2.5781]), and lower ψ (prior belief of burst) than the HSI group (95% HDI [−0.0128, −0.0025]). Other parameters, including ξ, τ, and ρ, were not significantly different between the two groups (see Details in Table 2).

**Table 2.** Differences between the SA and HSI groups on five parameters of BART’s EW model

Group parameter	SA	HSI	*t* 95%HDI
ρ (risk preference)	0.073 ± 0.008	0.065 ± 0.002	[−0.0145, 0.0386]
λ (loss aversion)	13.680 ± 0.349	12.222 ± 0.047	[0.3608, 2.5781] *
ψ (prior belief of explode)	0.065 ± 0.002	0.073 ± 0.0005	[−0.0128, −0.0025] *
ξ (updating exponent)	1.187e-04 ± 2.165e-05	8.034e-05 ± 0.0001	[0, 1e-04]
τ (inverse temperature)	106.328 ± 28.741	136.352 ± 51.209	[−106.2113, 41.0938]

Abbreviations: SA, Suicidal Attempt group; HIS, High suicidal ideation group.

*representing a significant difference between the SA and HSI groups.

### Resting-state functional connectivity

**Figure 1.**
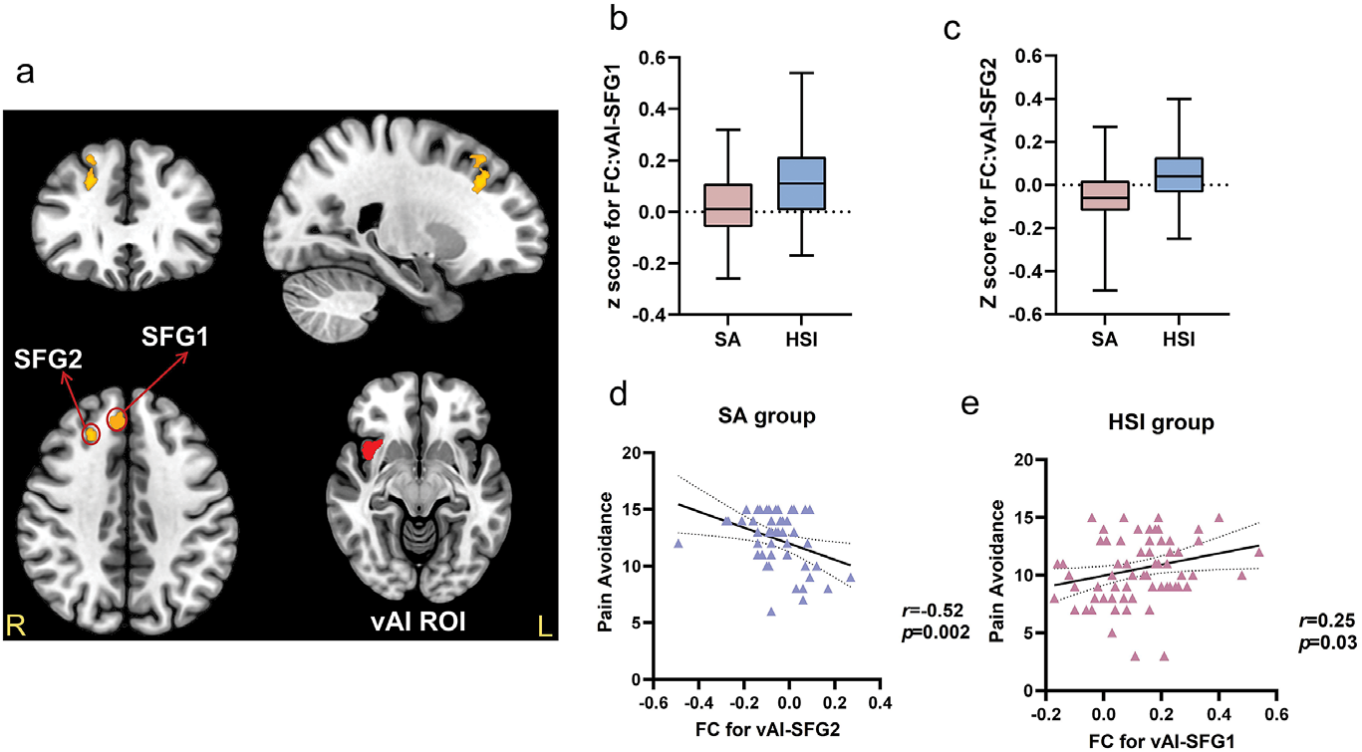
**Significant differences between SA and HSI groups in FC:vAI-SFG.** (a) Based on the right vAI ROI, the FCs between vAI and the two brain clusters within the SFG were significantly different between the SA and HSI groups. For the first cluster (SFG1): peak MNI coordinate: 10, 38, 36; *p* = 0.010, FDR cluster-level corrected; For the second cluster (SFG2): peak MNI coordinate: 24, 28, 32; *p* = 0.017, FDR cluster-level corrected. (b) The box plots displayed the FC-transformed Z-values between vAI and SFG1 in the SA and HSI groups. (c) The box plots displayed the FC-transformed Z-values between vAI and SFG2 in the SA and HSI groups. (d) Significant correlation between psychological pain avoidance and FC of vAI-SFG2 in the SA group with the suicide ideation of the current week and the worst time as covariates. **e.** Significant correlation between psychological pain avoidance and FC of vAI-SFG1 in the HSI group.

**Figure 2.**
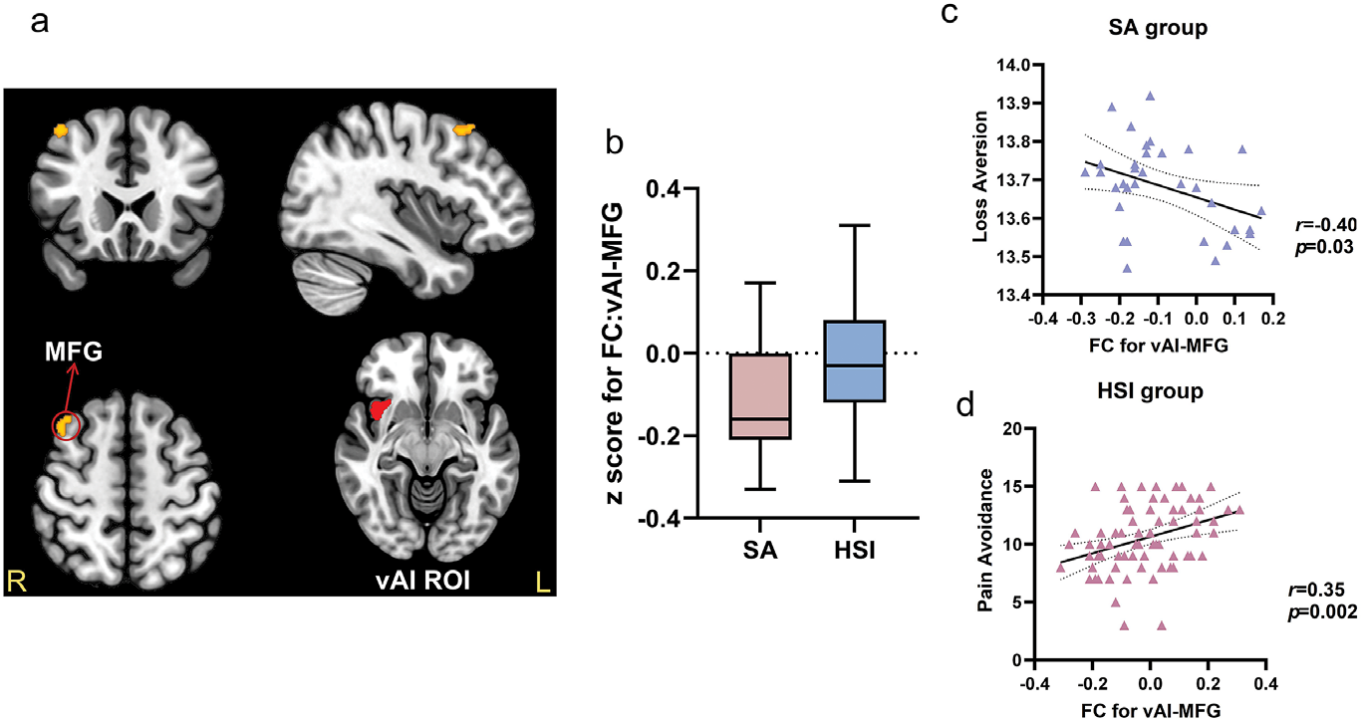
**Significant differences between SA and HSI groups in FC:vAI-MFG.** (a) Based on the right vAI ROI, the FC between vAI and MFG was significantly different between the SA and HSI groups (MFG: peak MNI coordinate: 42, 22, 54; *p* = 0.027, FDR cluster-level corrected) (b) The box plots displayed the FC-transformed Z-values between vAI and MFG in the SA and HSI groups. (c) Significant correlation between FC of vAI-SFG and loss aversion in the SA group with the suicide ideation of the current week and the worst time as covariates. (d) Significant correlation between FC of vAI-SFG and psychological pain avoidance in the HSI group.

**Figure 3.**
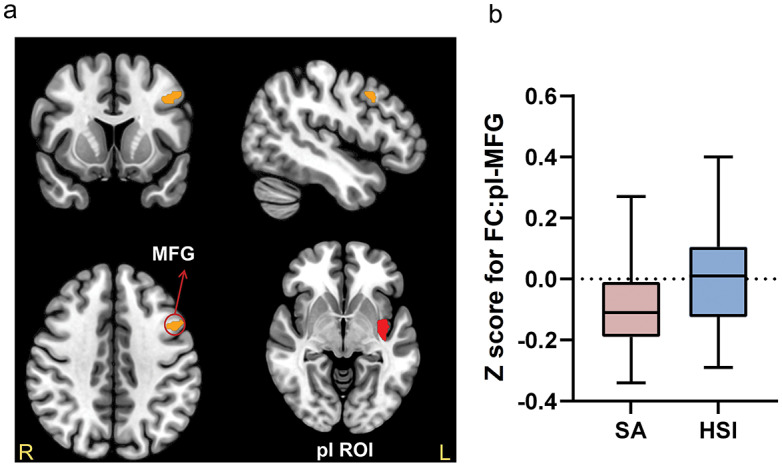
**Significant differences between SA and HSI groups in FC:pI-MFG.** (a) Based on the left pI ROI, the FC between pI and MFG was significantly different between the SA and HSI groups (MFG: peak MNI coordinate: −42, 12, 36; *p* = 0.002, FDR cluster-level corrected) (b) The box plots displayed the FC-transformed Z-values between pI and MFG in the SA and HSI groups.

As shown in Table 3, there are two significant FC differences found in the insula ROI. First, the SA group showed lower FC between the right ventral anterior insula (vAI) region and the right superior frontal gyrus (SFG) (FC:vAI-SFG) (Figure 1a,b,c), and between the vAI and the right middle frontal gyrus (MFG) (FC:vAI-MFG) (Figure 2a,b) when compared to the HSI group. Second, the SA group showed lower FC between the left posterior insula (pI) region and the left MFG (MFG) (FC:pI-MFG) (Figure 3) when compared to the HSI group (all p < 0.05, FDR corrected). There was no significant between-group FC difference in the dAI or the dACC ROI (results at p < 0.01 (uncorrected) are provided in Supplementary Table S4.).

**Table 3.** Significant differences in functional connectivity between the SA group and HSI group based on the vAI and pI ROIs

ROI	R/L	Brain region	BA	Cluster size (voxels)	MNI coordinate			Strength		t
					x	y	z	SA	HSI	
vAI	R	SFG R	9	68	10	38	36	0.02	0.12	−4.63
		SFG R	8	54	24	28	32	−0.06	0.05	−4.84
		MFG R	8	45	42	22	54	−0.11	−0.02	−4.17
pI	L	MFG L	9	90	−42	12	36	−0.10	0.01	−4.51

*Note: p < 0.001 for uncorrected voxel levels, and p < 0.05 for False Discovery Rate (FDR)-corrected cluster levels.*

Abbreviations: ROI, Region of Interest; R, Right; L, Left; BA, Broadman Area; vAI, ventral Anterior Insula; SFG, Superior Frontal Gyrus; pI, posterior Insula; MFG, Middle Frontal Gyrus.

### Results of correlation analysis

Only BART parameters, psychological variables, and the FC that showed a significant difference between the HSI and SA groups were selected for the correlation analyses. For BART parameters, the λ (*r* = −0.40, *p* = 0.03)(Figure 2c) and ψ (*r* = −0.39, *p* = 0.03) were both negatively correlated with the FC between the vAI and the MFG in the SA group Figure 2c.

For psychological variables, the FC between the vAI and the SFG was negatively correlated with psychological pain avoidance in the SA group (*r =* −0.52, *p* = 0.002) (Figure 1d), while positively correlated in the HSI group (*r =* 0.25, *p* = 0.03) (Figure 1e). Furthermore, the FC between vAI and the MFG was also positively correlated with psychological pain avoidance in the HSI group (*r* = 0.35, *p* = 0.002) (Figure 2d).

To further understand the evolution progress of suicidal ideation into action, we further did the correlation analysis between those FC and the suicidal ideation in the SA or HSI group, respectively. The results revealed FC of pI-MFG correlated with BSI-C and the FC of vAI correlated with BSI-W in the HSI group, while only the FC of pI-MFG correlated with BSI-W in the SA group. The detailed results were shown in the Supplementary Table S5.

## Discussion

The present study explored possible neuropsychological mechanisms related to the transition from suicide ideation to action by comparing FC of rs-fMRI and other psychological features between participants who had only suicide ideation (HSI group) and those with suicide ideation and attempt (SA group). At the neural mechanism level, the SA group showed significant differences in the FC for vAI-SFG, vAI-MFG, and pI-MFG when compared to the HSI group. At the psychological level, motivation to escape psychological pain, loss aversion, and prior belief of explode distinguished the SA group from the HSI group. In conjunction with the findings of the correlation between FCs and BART parameters, psychological variables, as well as the assessment of suicidal ideation, we explore and propose further hypotheses related to the neural mechanisms underlying the transition from suicide ideation to action.

### Pain avoidance and risk decision-making

Our study’s finding that the SA group had higher psychological pain avoidance than the HSI group is consistent with those reported by Li and colleagues [25, 49]. In terms of risk decision-making, our finding that the SA group had greater loss aversion than the HSI group is similar to those from Liu and colleagues using similar methodology [44], and others employing different decision tasks or models [26, 50]. Furthermore, the SA group showed a lower estimation of the probability of balloon explosion than the HSI group, which is consistent with the results of Liu et. al [44]. One possible reason for this is that the SAs tend to underestimate the negative effects of failed decision-making [51], and therefore underestimate the negative effects of suicide when they decide whether to commit suicide – for example, one could end up with major physical disability if the suicide fails. This belief bias may reduce their fear of suicide and propel them to take their own lives. Notably, participants in the SA group had all experienced suicide attempts without serious consequences (e.g., physical disability). Whether this experience of surviving past attempt(s) contributed to the “optimism” about balloon burst, or there were some other pre-existing related factors that helped to shift from suicidal ideation to suicidal action is unclear. More research is needed.

Our study also shows a significant inter-correlation between psychological pain avoidance and loss aversion (*r* = 0.31, *p* = 0.004), and initial belief of burst (*r* = −0.30, *p* = 0.005), reproducing a prior finding [44]. Daniel and colleagues reported that individuals who are ready to commit suicide have a tendency to exaggerate their perceived losses and minor losses are perceived as being so overwhelming that they reinforce their suicide behavior [52]. In addition, when individuals live in great psychological pain, it may escalate their tendency to avoid such aversive situations, as Milner and colleagues have found that suicide attempters showed more active avoidance tendencies when confronted with uncertainty in aversion situations [53]. This tendency to exaggerate their perceived loss may further lead people to view life as a series of losses and underestimate the downside of suicide. We believe that the interplay between the motivation to avoid psychological pain, the aversion to loss, and the underestimation of the downside of suicide could result in the counterintuitive “optimism” and ultimately heighten the likelihood of transition from suicidal ideation to action.

### Potential neural mechanisms underlying transition from suicide ideation-to-action

Comparing FC between the SA and HSI groups, our study found two novel and distinct neural pathways of interest related to the insula: (i) a decreased FC between the vAI and the SFG, and between the vAI and the MFG – insula areas responsible for the regulation of emotions and optimal decision-making, among other functions; and (ii) a decreased FC between the pI to the MFG – insula regions related to interoceptive functions.

The insula is complex, known to be a multimodal region that integrates information from internal and external states and plays an important role in the processing of emotions and cognition [54]. The current study implicates two of the three functionally distinct regions, including vAI – involved in emotion processing, and pI – responsible for interoceptive processing (i.e., producing a sense of the internal state of the body based on the integration of internal and external physiological signals) [55, 56]. Previous research on suicide ideation-to-action involving the insula has been limited but informative. Two such studies have used a paradigm related to decision-making and found abnormal insula activity in suicide attempters during their risk-taking tasks [50, 57]. In addition, one study compared the FC based on the insula as ROI in the resting state between patients with MDD with and without suicide attempt history and found abnormal FC of the pI with the orbitofrontal cortex and a series of motor cortices [58]. Although these studies all broadly implicated the insula, the mechanism of how it mediates the transition from suicide ideation to action is unclear. The present study advances the current understanding via our new findings involving the subregions of the insula that could allow inference on how they might play a role through the specific functions these subregions play.

Firstly, we explore the findings that a lower resting-state FC between the vAI and the SFG (and the MFG) distinguished the SA group from the HSI group. Based on the concept that inter-group differences may point to potential mechanisms involved in the ideation–to-action transition, we surmise that the vAI is strongly implicated in such. This hypothesis is further buttressed by our findings on two psychological factors that also differentiated suicide attempters from ideators – one being that the FC: vAI-SFG was negatively correlated with psychological pain avoidance in the SA group (while positively correlated in the HSI group), and secondly the FC:vAI-MFG was negatively correlated with loss aversion in the SA group. Based on the knowledge that the vAI is generally involved in emotion processing, and has a specific role in the processing of pain [59], and that the SFG and MFG are neuroanatomically associated with the dorsolateral prefrontal cortex (DLPFC), which is thought to be the key hub that processes the top-down control of emotion, cognition, and decision-making [60, 61], our findings may suggest that dysfunctions in these region-specific roles, involving the vAI in common, contribute to the transition from suicide ideation-to-action.

In addition, the vAI is an important brain area of the somatic brain system for integrating the physiological state of the body and can further transmit information to the prefrontal cortex [62]. According to the somatic marker hypothesis [63], the link between emotions and specific objectives or perceptions may be marked in a specific brain system, which leads to faster responses to specific targets and sometimes it only changes in the brain neurotransmitter system without actual physical changes (an “as if body loop” mechanism). Thus, repeated psychological pain may lead individuals to respond more quickly to stimuli that produce psychological pain, become less intolerant of psychological pain, and develop more motivation to avoid psychological pain. When participants in the HSI group repeatedly experienced psychological pain, more top-down regulation from the SFG and MFG was needed to manage an increased motivation to avoid psychological pain. In support of our assertion, research has also found higher DLPFC activation during active regulation of negative emotional scenes in adolescents with suicidal ideation [64], suggesting that suicide ideators may require more top-down emotional regulation from the DLPFC to regulate their negative emotions – such as psychological pain.

With experiencing psychological pain repeatedly, the related somatic marker in vAI gradually becomes stabilized in the HSI group. When vAI-related FCs become stabilized impairments, it will unconsciously influence the decision-making process for suicide according to the somatic marker hypothesis [63], with a greater tendency to view living as a loss and underestimate the downside of suicide. To demonstrate it, we recalculated the correlation between vAI-SFG and psychological pain avoidance after matching the psychological pain avoidance levels of the two groups, and this positive-to-negative switch (HSI vs SA) in the correlation pattern still exists, further indicating vAI-SFG is stabilized impairment in SA group, and not due to different levels of psychological pain avoidance in the two groups. Furthermore, the trait impairments of vAI-related FCs in the SA group would be further intensified when undergoing more psychological pain avoidance. Overall, we hypothesize that the shift from positive to negative correlation patterns may represent a shift from state to trait impairment of vAI and its specific circuits linked to SFG and MFG, which further mediate the shift from suicidal ideation to action through impaired top-down emotion and decision-making regulation.

Secondly, we explore the findings that the SA group showed a lower FC between pI and the MFG than the HSI group. This diminished connection may suggest that suicide attempters may experience blunted interoception processing, with less optimal integration of internal and external signals [55, 56]. In suicide attempters, this blunted sensitivity to bodily signals may be related to an increased tolerance to aversive sensations or reduced aversion to physical threats, therefore escalating suicide capacity and further increasing the probability of transitioning from suicide ideation to action. To illustrate, one study has found that suicide attempters, when compared to controls had reduced activation of the mid/posterior insula during their attention to heartbeat sensation tests; also, suicide attempters can endure longer breath-holding and cold temperature challenges, as well as having lower accuracy in heartbeat perception, when compared to non-suicide attempters [65]. The authors hypothesized that the “interoceptive numbing,” characterized by increased tolerance for aversive sensations and decreased awareness of non-aversive sensations is implicated in suicide behavior, and associated with the posterior insula. Our current study results support this theory.

Lastly, with regard to correlations between FC of pI-MFG and suicidal ideation, we found differences between the SA and HSI groups- the former is correlated with suicidal ideation at the worst time, and the latter is correlated with suicide ideation at the current week. One possible explanation for this difference is that suicide attempts may typically occur at the “worst” psychologically distressed time in the SA group. According to the suicide capacity theory proposed by Klonsky [66], suicide capacity could be elevated through repeated experiences of aversive stimuli. Hence, the FC for pI-MFG may be altered by repeated experiences of aversive stimuli (e.g., suicide attempts) and lowering the threshold for the transition from suicidal ideation to action. Instead, the individuals in the HSI group do not typically experience severe physical injuries like those in the SA group, their internal perception becomes increasingly disrupted as they encounter more and more aversive events, which may explain why their FC for pI-MFG correlated with suicidal ideation during the current week. In addition, we found no significant correlation between FC for vAI-MFG and vAI-SFG with suicidal ideation in the SA group, while we found a significant correlation in the HSI group. In line with the somatic marker hypothesis, FC of vAI-SFG and vAI-MFG are still in the process of formation and have not yet stabilized for individuals in the HSI group, thus always associated with suicidal ideation in the worst period. Overall, vAI, PI, and their associated functional connectivity appear to represent two distinct functions – the former may be trait-specific and the latter state-specific neuro biomarkers, and both of these neuro biomarkers lead to an increased risk of subsequent suicide actions.

## Limitations

The current study has a number of limitations. First, we did not include a healthy control group, and thus we are unable to provide a “baseline” on suicidal ideation and suicidal attempt issues. Nevertheless, the inclusion of a healthy control group yielded perplexing disparities between the SA, HSI, and the healthy control group. Those discrepancies raised questions including depression levels, with/without suicidal ideation, and with/without suicidal action as contributing factors. Previous studies that employed a healthy control group and found similar general results as ours may help to compensate for this limitation to some extent [67, 68].

Second, our cross-sectional design may make it difficult to determine whether the findings in this study are “traits” or “states,” and more longitudinal studies are needed. Third, although we think the resting state during the scanning is similar to the state that people consider whether to commit suicide, the assumption may introduce biases. Future studies could attempt to guide subjects to think about suicide during the scanning process under ethical guidance. Finally, we had a relatively small sample size for the behavior tasks, and a larger sample size would improve this in future studies.

## Conclusion

The present study combined rs-fMRI, the BART behavior task, and other salient measures to explore the potential neuropsychological mechanisms underlying the transition from suicidal ideation to action by comparing MDD patients who have high suicidal ideation with and without a history of suicide attempts. We found two distinct neural mechanisms involving the insula. One such neural mechanism is related to the abnormal FC of vAI with SFG and MFG, which might influence the top-down regulation of emotion and decision-making process enabling the transition from suicidal ideation to action. The second neural mechanism is related to the abnormal FC of pI with MFG, which may lead to a lowered threshold for suicide by blunting the perception of physical harm. Overall, this study provides empirical evidence that the insula may play an important role in the transition from suicidal ideation to action and support the “tentative brain circuitry model” of suicidal thoughts and behaviors as proposed by Schmaal and colleagues.

## Supporting information

Fang et al. supplementary material Fang et al. supplementary material

Fang et al. supplementary material Fang et al. supplementary material

## Supplementary material

The supplementary material for this article can be found at http://doi.org/10.1192/j.eurpsy.2023.2444.
