# Supplementary material for: Potential neuropsychological mechanism involved in the transition from suicide ideation to action – a resting-state fMRI study implicating the insula
Source: Eur Psychiatry. 2023 Sep 11;66(1):e69. doi: 10.1192/j.eurpsy.2023.2444 (PMC10594382; doi:10.1192/j.eurpsy.2023.2444)
Supplement: Fang et al. supplementary material [file S0924933823024446sup001.docx]

**Supplementary materials**

**Method**

**Detailed methodology for suicide attempts in the SA group**

Table S1 Detailed methodology for suicide attempts

| Subject | Frequency | Methods |
| --- | --- | --- |
| 1 | 2 | jumping from a building + traffic collision |
| 2 | 3 | wrist cutting +hit the wall +jumping from a building |
| 3 | 2 | jumping from a building +overdose of medication |
| 4 | 1 | overdose of medication |
| 5 | 2 | wrist cutting +overdose of medication |
| 6 | 3 | Overdose +hanging +wrist cutting |
| 7 | 1 | wrist cutting |
| 8 | 1 | wrist cutting |
| 9 | 1 | overdose of medication |
| 10 | 1 | wrist cutting |
| 11 | 1 | jumping from a building |
| 12 | 1 | wrist cutting |
| 13 | 2 | jumping to a river + wrist cutting |
| 14 | 1 | overdose of medication |
| 15 | 1 | overdose of medication |
| 16 | 1 | wrist cutting |
| 17 | 1 | Burning charchoal |
| 18 | 1 | overdose of medication |
| 19 | 3 | wrist cutting+ overdose of medication + jumping from a building |
| 20 | 2 | wrist cutting + jumping from a building |
| 21 | 1 | traffic collision |
| 22 | 1 | wrist cutting |
| 23 | 3 | Immersion +wrist cutting + jumping from a building |
| 24 | 1 | wrist cutting |
| 25 | 1 | wrist cutting |
| 26 | 2 | jumping from a building + overdose of medication |
| 27 | 1 | jumping from a building |
| 28 | 1 | wrist cutting |
| 29 | 1 | wrist cutting |
| 30 | 1 | wrist cutting |
| 31 | 2 | jumping from a building + wrist cutting |
| 32 | 1 | hanging |
| 33 | 1 | wrist cutting |
| 34 | 2 | wrist cutting + overdose of medication |
| 35 | 1 | jumping from a building |
| 36 | 1 | jumping from a building |
| 37 | 2 | jumping from a building + jumping to a river |
| 38 | 1 | wrist cutting |
| 39 | 1 | overdose of medication |
| 40 | 2 | overdose of medication + wrist cutting |
| 41 | 1 | traffic collision |
| 42 | 2 | jumping from a building + wrist cutting |
| 43 | 1 | overdose of medication |
| 44 | 1 | wrist cutting |
| 45 | 1 | jumping from a building |
| 46 | 2 | overdose of medication + jumping to a river |
| 47 | 1 | overdose of medication |

**The Balloon Analogue Risk Task**

As shown in Figure S1, at the beginning of this task, a virtual balloon is represented on the screen, with one indicator showing the participant's reward counts of the current trial and another on rewards gained from the total trials. Participants are guided to make a series of risk decisions to obtain maximum rewards by choosing to press button 1 to inflate, or button 2 to discontinue inflation. In the process of pressing button 1, the balloon grows larger, accompanied by the constant possibility of obtaining bigger rewards versus explosions. If the balloon explodes during inflation, the screen will show “You lose!” and the rewards of the current trial will be deducted from the total reward. On the other hand, when participants press button 2 and the balloon will discontinue inflation, the screen will show “You win!”, and rewards of the current trial will be added to the total reward.


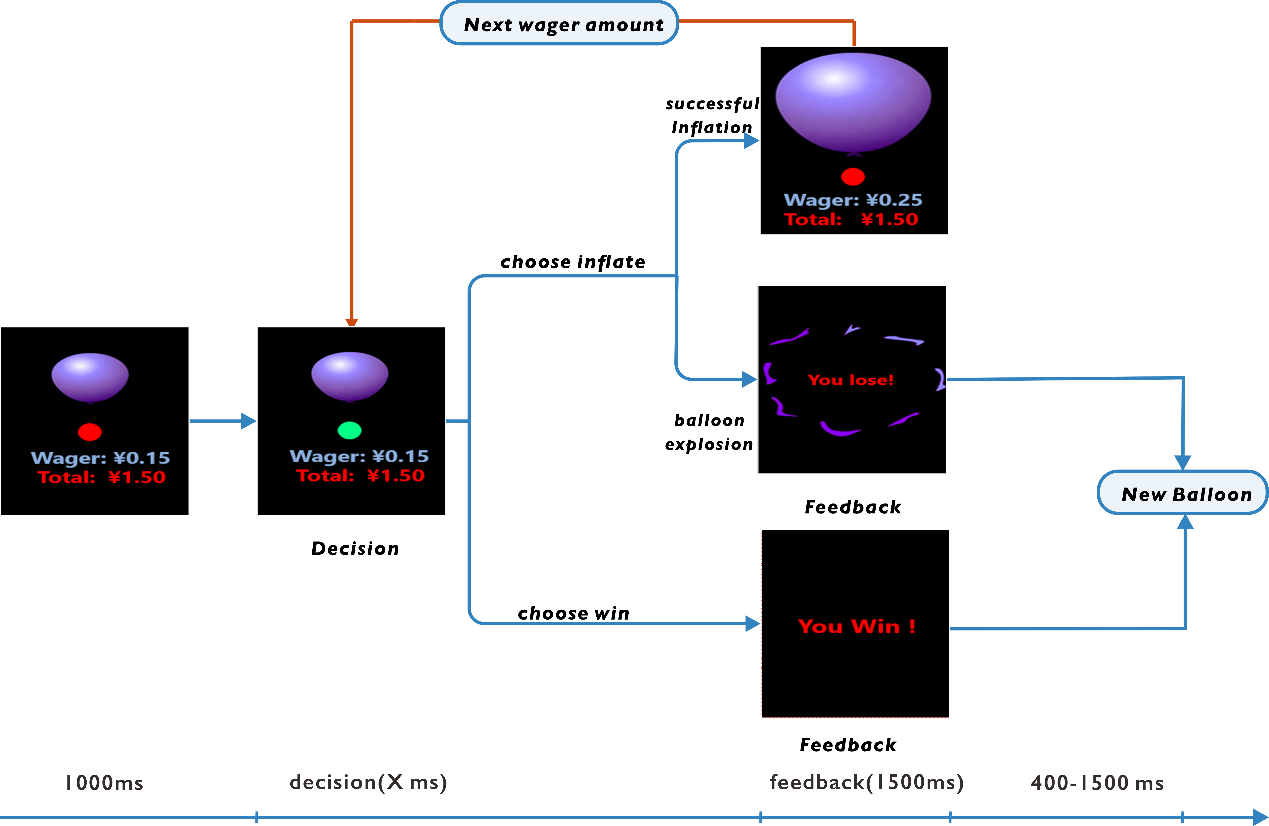


**Figure S1. Overview of BART.** Each time the green circle lights up, the participant selects to inflate the balloon or stop inflating it. As the balloon continues to inflate, a reward corresponding to the balloon size was granted and shown in “wager”. If the balloon exploded, the cumulative wager amount will be deducted from the participant’s account. If the balloon did not explode, the cumulative wager amount will be added to the participant’s account. Following a win-outcome or a loss-outcome, the screen was blank, after which a new balloon appeared, and the inflation process repeated. BART, balloon analog risk task.

**Two sample t-tests between the retained sample and dropout sample of BART**

Of the 120 participants, 86 completed BART, and there were no significant differences in clinical measures between the retained and dropout samples, as shown in Table S2:

Table S2 Two sample t-test between retained and dropout sample

|  | Retained participants (n=86) | Dropped participants (n=34) | *X*^2^/t | *p* |
| --- | --- | --- | --- | --- |
| **Male/Female (n)** | 20/66 | 5/29 | 1.08 | 0.30 |
| **Age (years)** | 20.88±4.98 | 22.44±5.69 | 1.48 | 0.14 |
| **Education (years)** | 13.23±2.72 | 13.79±2.64 | 1.03 | 0.31 |
| **Duration of illness (months)** | 15.54±15.92 | 15.39±16.00 | 0.05 | 0.96 |
| **First episode/non-first episode** | 74/12 | 29/5 | 0.01 | 0.92 |
| **History of suicide attempt (with/without)** | 33/53 | 14/20 | 0.08 | 0.78 |
| **BDI score** | 36.01±7.61 | 36.58±5.67 | 0.39 | 0.70 |
| **BSI_C score** | 13.00±7.17 | 14.44±7.17 | 0.99 | 0.32 |
| **BSI_W score** | 24.63±5.61 | 25.00±5.70 | 0.33 | 0.75 |
| **S_AI** | 61.24±9.94 | 60.28±12.34 | -0.42 | 0.67 |
| **T_AI** | 65.41±6.18 | 65.14±8.30 | -0.19 | 0.85 |
| **TDPPS score** | 65.28±9.88 | 66.47±7.46 | 0.64 | 0.53 |

*Note:* BDI, Beck Depression Inventory; BSI_C, Beck Scale for Suicide Ideation at the current time; BSI_W, Beck Scale for Suicide Ideation at the worst time; SAI: State anxiety inventory; TAI: Trait Anxiety Inventory; TDPPS: Three-dimensional Psychological Pain Scale.

**Exponential Weight Updating model (EW model)**

The exponential weight updating model is built based on the four-parameter model of BART, which has shown good prediction performance and parameter recovery in suicide-related samples. EW model is based on two hypotheses: the first one is that participants have an initial belief about the probability of exploding and update the belief after the observation of each trial; The second one is participants decided whether or not to continue pumping the balloon before every press, instead of estimating an optimal number of pumps before each trial.

The $p_{k}^{burst}$ represents the probability of exploding on the k trial, ψ represents the initial belief about the probability of exploding, the $p_{k-1}$presents the observed probability of exploding on the k-1 trial. Then, the weight of ψ is represented by $\omega_{k-1}$, and the weight of $p_{k-1}$ is represented by ($1-\omega_{k-1})$. Hence, based on the first hypothesis, the $p_{k}^{burst}$ is described as follows:

$p_{k}^{burst}=\omega_{k-1}\psi+(1-\omega_{k-1})p_{k-1}$ 0<ψ<1, η>0 (1)

Where the $p_{k-1}$ is defined as the ratio of the total number of pumps $\sum_{i=0}^{k-1} n_{i}^{pump}$ to the total number of pumps in case of an explosion$\sum_{i=0}^{k-1} \left( n_{i}^{pump}-n_{i}^{success} \right)$:

$p_{k-1}=\frac{\sum_{I=0}^{n-1} \left( n_{i}^{pump}-n_{i}^{success} \right)}{\sum_{i=o}^{k-1} n_{i}^{pump}}$ (2)

The participants will gain more experience as the number of pumps increases, and the subjective probability of exploding would be less dependent on the initial belief. Hence, the $\omega_{k-1}$ depends on the total number of pumps $(\sum_{i=0}^{k-1} n_{i}^{pump})$and the update rate of experience ($\xi$). A higher $\xi$ represents participants' difficulty learning from experience and more tend to focus on the current outcomes. When $\xi$→∞, the decisions of the participant were almost entirely determined by the outcome of the current decision. Thus, $\omega_{k-1}$ is described as follows:

$\omega_{k-1}=e^{-\xi\sum_{i=0}^{k-1} n_{i}^{pump}}$ (3)

And the $p_{k}^{burst}$ can be described as follows

$p_{k}^{burst}=e^{-\xi\sum_{i=0}^{k-1} n_{i}^{pump}}\psi+(1-e^{-\xi\sum_{i=0}^{k-1} n_{i}^{pump}})p_{k-1}$0<ψ<1, $\xi$>0 (4)

To address the second hypothesis, we utilized the prospect theory[1] and defined the ($U_{kl}^{pump}$) represents the the subjective utilities for inflating and not-inflating a balloon on trial k for pump $l$ press l. And the ($U_{kl}^{pump}$) can be described as follows:

$U_{kl}^{pump}=\left( 1-p_{k}^{burst} \right)r_{l}^{\rho}-p_{k}^{burst}\lambda\{{\left( l-1 \right)r\}}^{\rho}$ 0<ρ<2, λ>0 (5)

$U_{kl}^{transfer}=0$ (6)

Where $\lambda$ is loss aversion, $r$ is the amount of value that the participant can practically gain or lose, and *p* is risk preference.

Then, the probability of pumping the balloon on trial on trial *k* for pump *l* is described as follows:

$p_{kl}^{pump}=\frac{1}{1+e^{\tau\left( U_{kl}^{transfer}-U_{kl}^{pump} \right)}}$ τ≥0 (7)

In Equation (7), τ is inverse temperature, which means how deterministic or random the choice is. The lower the τ, the more random the selection. Five parameters are finally obtained in the EW model: ψ(prior belief of explode), $\xi$ (updating exponent), $\rho$(risk aversion), $\lambda$(loss aversion), τ(inverse temperature).

**Scanner parameter**

**Field map images**：Repetition time: 8.6ms; echo time: 4.0ms; FOV:250mm; flip angle: 20°; voxel size: = 0.488×0.488×0.488 mm^3^

**BOLD Resting State images：** Shot gradient echo planer imaging, single shot GRE-EPI sequence was used. Repetition time: 2000ms; echo time: 30ms; FOV:220mm; flip angle: 90°; voxel size: 3.44×3.44 ×4 mm^3^; matrix=64×64; slice thickness=4.0mm. A successive of 240 brain volumes were obtained within 8 minutes. Then, the scanner parameter was optimized: the Repetition time was added 500ms, the slice thickness was reduced 0.6mm, and the voxel size was changed to 3.4×3.4×3.4 mm^3^. A successive 200 brain volumes were finally acquired within 8.29 minutes.

**High-resolution coplanar anatomical images:** Magnetization prepared rapid gradient echo (MPRAGE) sequence was used. Repetition time: 2000ms; echo time: 3.01ms; FOV:256mm; flip angle: 9°; voxel size: 1×1×1 mm^3^; matrix=256×256; slice thickness=1.0mm. A successive of 176 brain volumes were obtained within 8.8 minutes. Then, the scanner parameter was optimized: the Repetition time added 1000ms, the voxel size was changed to 0.8×0.8×0.8 mm^3^. A successive of 224 brain volumes were finally acquired within 8.35 minutes.

Of the total of 120 participants, 47 participants were scanned using the initial scanner parameter, and 73 participants were scanned using the optimized scanner parameter. There was no significant difference in the proportion of scanner parameter types in the two groups (SA: 22/25, HSI:27/46, χ2=1.14, *p*=0.23).

**Imaging preprocessing**

The imaging data were preprocessed using the conn toolbox (CONN 20b, HTTPS:// conn-toolbox.org/) implemented in MATLAB R2018a and applying standard preprocessing steps:(a) The first 6 time points were discarded to avoid effects of signal disequilibrium and subject maladjustment; (b) The middle slice was chosen as a reference to conduct the slice time correction; (c) Realignment correction was conducted by discarding time points that had translation≥ 2 mm or rotation ≥ 2◦ from the time series; (d) In the normalization step, firstly, individual structural images were co-registered with the mean functional image; then the transformed structural images were segmented into white matter, gray matter, and cerebrospinal fluid, and further normalized to the Montreal Neurological Institute (MNI) space with resampling into a 2-mm cubic voxel; (e) All data were smoothed with a 6-mm full-width at half-maximum (FWHM) Gaussian kernel; (f) The data obtained from the above steps were regressed in a linear model, including Friston-24 motion parameters[2], white matter and cerebrospinal fluid; and (g) The bandpass filter was conducted with a frequency range of 0.01 to 0.08 Hz.

**Results**

**Diagnostics for MCMC**

We generated four independent Markov chain Monte Carlo (MCMC) chains, each consisting of 2000 iterations, resulting in a total of 8000 samples. To ensure the stability of the chains, we discarded the first half of each chain, commonly known as "burn-in." Subsequently, we retained the first 1000 samples of each of the four chains and further analyzed the remaining 4000 samples.

To assess the quality of the MCMC samples, we performed the Gelman-Rubin test, which calculates the potential scale reduction factor (R̂) for each group. The R̂ for SA and HSI groups is shown in Table S3.

Table S3 Statistics of posterior distributions of group parameters for the SA and HSI group

| Group parameter | SA | HSI |
| --- | --- | --- |
| ρ (risk preference) | 1.01 | 1.01 |
| λ (loss aversion) | 1.02 | 1.01 |
| ψ (prior belief of explode) | 1.02 | 1.01 |
| ξ (updating exponent) | 1.01 | 1.01 |
| τ (inverse temperature) | 1.01 | 1.01 |

*Note:* Ȓ is the convergence value of the chain, for all parameters, Ȓ values < 1.1 indicate that the chains converged to the target distribution.

**Uncorrected results for functional connectivity based on dAI and dACC ROIs**

We computed the original whole-brain difference comparisons in the dACC and dAI with p uncorrected<0.01. In terms of dACC, as shown in Table S4, the FC between dACC_L and Paracingulate Gyrus_R was lower in the SA group than in the HSI group, and the FC between dACC_L and Cerebelum_R was higher than in the HSI group. However, there was still no difference between groups in terms of dAI ROI.

Table S4 Results of functional connectivity based on dAI ROI

| ROI | R/L | Brain Region | BA | Cluster size  (voxels) | MNI coordinates | | | t |
| --- | --- | --- | --- | --- | --- | --- | --- | --- |
|  |  |  |  |  | x | y | z |  |
| dACC | L | Paracingulate Gyrus_R | 10 | 34 | 12 | 48 | 06 | -4.43 |
| dACC | R | Cerebelum_R |  | 28 | 36 | -84 | -28 | 4.43 |

*Note:* dACC, dorsal anterior cingulate cortex; L, left; R, right; BA, Brodmann Area; *p_uncorrected_<*0.01.

**The correlation between FCs and suicidal ideation**

We conducted Pearson’s correlation analysis between FCs and the BSI-C (the current week’s suicidal ideation) and BSI-W (the worst time’s suicidal ideation), the detailed results were shown in Table S5:

Table S5 The Pearson’s correlation between FC and suicidal ideation

| FC | BSI-C | | | | BSI-W | | | |
| --- | --- | --- | --- | --- | --- | --- | --- | --- |
|  | *SA group* | | *HSI group* | | *SA group* | | *HSI group* | |
|  | *r* | *p* | *r* | *p* | *r* | *p* | *r* | *p* |
| vAI_SFG1 | -0.60 | 0.67 | 0.15 | 0.21 | 0.11 | 0.48 | **0.26** | ***0.03*** |
| vAI_SFG2 | 0.18 | 0.24 | 0.07 | 0.56 | 0.16 | 0.29 | 0.19 | 0.11 |
| vAI_MFG | 0.02 | 0.91 | 0.19 | 0.11 | 0.10 | 0.51 | **0.26** | ***0.03*** |
| pI_MFG | 0.27 | 0.07 | **0.40** | ***<0.01*** | **0.32** | **0.03** | 0.10 | 0.39 |

*Note:* The coordinate for SFG1 is (10, 38, 36) and the coordinate for SFG2 is (24, 28, 32).

vAI, ventral Anterior Insula; SFG, Superior Frontal Gyrus; pI, posterior Insula; MFG, Middle Frontal Gyrus. Significant correlation coefficients are shown in bold italics.

References:

1. Kahneman, D. and A. Tversky, Prospect Theory: An Analysis of Decision under Risk. Social Science Electronic Publishing, 1979.

2. Friston, K., Movement-related effects in fMRI time-series. Magnetic Resonance in Medicine, 2015. 35.
